# Adipose Tissue Expandability as a Link Between Nutrition and Obesity-Related Metabolic Risk: Insights from Bariatric Surgery

**DOI:** 10.3390/nu18183075

**Published:** 2026-09-20

**Authors:** Emilia Jiménez-Flores, Claudia Reytor-González, Dolores Jima Gavilanes, Gianluca Rossetti, Vincenzo Pilone, Luca Parrillo, Michela Cavallo, Luigi Cobellis, Daniel Simancas-Racines, Luigi Schiavo

**Affiliations:** 1 Center for Evidence Ecosystems, Implementation Science, and Decision-Making (CIDES), Facultad de Ciencias de la Salud y Bienestar Humano, Universidad Tecnológica Indoamérica, Ambato 180150, Ecuador; 2 Escuela de Medicina, Universidad Espíritu Santo, Samborondón 0901952, Ecuador; 3 General and Bariatric Surgery Unit, Abano Terme Policlinic, 35031 Padova, Italy; 4 Public Health Department, University of Naples Federico II, 80131 Naples, Italy; 5 Institute of Endotypes in Oncology, Metabolism and Immunology (IEOMI), National Research Council, 80131 Naples, Italy; 6 Department of Translation Medicine, Federico II University of Naples, 80131 Naples, Italy; michela.cavallo@unina.it; 7 Unit of General Surgery, Casa Di Cura “Prof. Dott. Luigi Cobellis”, 84078 Vallo della Lucania, Italy; 8 Department of Medicine, Surgery and Dentistry “Scuola Medica Salernitana”, University of Salerno, 84081 Baronissi, Italy

**Keywords:** adipose tissue expandability, adipocyte hypertrophy, hyperplasia, lipid spillover, ectopic fat deposition, insulin resistance, type 2 diabetes, adipose tissue fibrosis, chronic inflammation, lipotoxicity

## Abstract

Adipose tissue expandability refers to the capacity of white adipose tissue to store excess energy safely while preserving metabolic homeostasis. This adaptive response takes place via both an increase in adipocyte number (hyperplasia) and size (hypertrophy). Healthy expansion requires coordinated angiogenesis, extracellular matrix remodeling, and functional adipose stem and progenitor cells. Within this conceptual framework, when expandability is limited—due to genetic factors, aging, or chronic overnutrition—adipocyte hypertrophy predominates, which is hypothesized to trigger hypoxia, endoplasmic reticulum stress, chronic low-grade inflammation, and fibrosis. These changes are proposed to form a physical and metabolic barrier that compromises further safe lipid storage, potentially driving the spillover of surplus lipids to ectopic sites: the liver (promoting steatosis and hepatic insulin resistance), skeletal muscle (intramyocellular lipid accumulation and impaired glucose uptake), and pancreas (β-cell dysfunction and reduced insulin secretion). Lipotoxic intermediates such as diacylglycerols and ceramides activate protein kinase C isoforms and may impair insulin receptor signaling, thereby contributing to systemic insulin resistance. The clinical correlate is a spectrum of metabolic disorders including type 2 diabetes, dyslipidemia, metabolic dysfunction-associated steatotic liver disease, and cardiovascular disease. Understanding the determinants of adipose tissue expandability—from adipose stem cell function and angiogenesis to fibrosis and immune cell infiltration—provides a framework for identifying individuals at risk despite normal body weight and for developing targeted therapies that restore healthy adipose tissue expansion. The aim of this narrative review is to analyze how nutritional factors shape adipose tissue expandability and how limitations in expandability drive obesity-related metabolic disease. To this end, we leverage bariatric surgery as a physiological model illustrating how weight loss reduces lipid overflow while intrinsic adipose tissue expandability may remain incompletely restored.

## 1. Introduction

The global obesity pandemic continues to challenge public health systems worldwide [1], yet the relationship between excess adiposity and metabolic disease remains incompletely understood [2]. A clinically relevant observation is that not all individuals with obesity develop metabolic complications; some maintain preserved insulin sensitivity and remain free from type 2 diabetes (T2D) and cardiovascular disease despite comparable body mass index (BMI) [2]. This phenotypic heterogeneity—commonly described as metabolically healthy versus unhealthy obesity (MHO vs. MUO)—suggests that total adiposity alone is insufficient to explain metabolic risk, highlighting the need to identify additional determinants of disease susceptibility.

The adipose tissue expandability hypothesis proposed by Sethi and Vidal-Puig provides a mechanistic framework to account for this variability. It postulates that metabolic dysfunction in obesity arises not merely from excess fat accumulation but from a limited capacity of subcutaneous adipose tissue (SAT) to safely expand in response to energy surplus [3]. When this storage capacity is exceeded—due to genetic, nutritional, or structural constraints such as fibrosis—lipids are increasingly redirected toward visceral and ectopic sites, including the liver, skeletal muscle, and pancreas. This redistribution is associated with lipotoxicity, impaired insulin signalling, and metabolic dysregulation [4]. Within this framework, individuals with preserved adipose expandability may preferentially store excess energy in SAT and therefore exhibit a more favorable metabolic profile, whereas those with restricted expandability are more prone to ectopic lipid deposition and associated complications [5]. However, several aspects remain unresolved, including the extent to which nutritional factors dynamically modulate expandability and whether impaired expandability can be modified or restored [6]. In addition, the interpretation of bariatric surgery—the most effective intervention for severe obesity—has largely focused on adipose tissue remodeling [7]. It is important to consider bariatric surgery as a physiological context that reveals the limits of adipose expandability under conditions of rapid and profound energy deficit.

In this context, while several existing reviews have addressed adipose tissue fibrosis, nutritional metabolism, or the clinical outcomes of bariatric surgery in isolation, the present manuscript offers a more integrative perspective by examining the dynamic interplay between nutritional determinants and the structural limits of adipose expandability. Furthermore, it leverages the profound weight loss induced by bariatric surgery as a model to distinguish between systemic metabolic recovery and the potential persistence of intrinsic tissue-level dysfunction. Accordingly, this narrative review aims to explore how nutritional factors, including energy balance, macronutrient composition, and dietary patterns, influence adipose tissue expandability, and how limitations in this capacity contribute to the development and progression of obesity-related metabolic disease.

## 2. Methods

This narrative review primarily considered publications available from database inception through early 2026. Literature searches were conducted in PubMed/MEDLINE, Cochrane Library, and Google Scholar using terms including “adipose tissue expandability”, “adipocyte hypertrophy”, “hyperplasia”, “lipid spillover”, “ectopic fat deposition”, “insulin resistance”, “type 2 diabetes”, “adipose tissue fibrosis”, “chronic inflammation”, and “lipotoxicity”. Eligible articles included original research, systematic reviews, meta-analyses, and narrative reviews addressing adipose tissue expandability in the context of bariatric surgery. Studies examining the molecular mechanisms of adipose expansion, nutritional determinants, and tissue-level remodeling were included, along with clinical trials and cohort studies evaluating metabolic outcomes post-bariatric surgery. Selected articles were analyzed to synthesize evidence on the interplay between dietary inputs, surgical lipid off-loading, and the structural limits of subcutaneous adipose tissue, establishing a conceptual framework for personalized metabolic risk stratification.

## 3. Adipose Tissue Expandability: Concept and Biological Foundations

### 3.1. Definition and Conceptual Framework

Adipose tissue expandability refers to the capacity of white adipose tissue (WAT) to store excess energy as triglycerides without compromising tissue function or systemic metabolic homeostasis. Under normal physiological conditions, WAT expands in response to chronic positive energy balance through two distinct but complementary mechanisms: hypertrophy (enlargement of pre-existing adipocytes) and hyperplasia (increase in adipocyte number via differentiation of adipocyte progenitor cells) [8]. The capacity of subcutaneous WAT to adapt to energy storage demands plays a crucial role in determining metabolic health. When this capacity becomes compromised—either due to reaching its storage limit or because of inadequate structural and vascular support—surplus lipids are redirected to ectopic tissues, where they can induce lipotoxic effects [9]. For instance, lipid accumulation in the liver and skeletal muscle contributes to insulin resistance, while deposition in pancreatic β-cells impairs insulin secretion [10,11,12]; concurrently, ectopic lipid storage in cardiac tissue is linked to cardiometabolic complications, and adipose tissue dysfunction promotes a state of chronic low-grade inflammation [13,14].

The expandability concept builds on the understanding that adipose tissue is not a passive energy reservoir but a highly dynamic endocrine organ. Its dysfunction is characterized by adipocyte hypertrophy, impaired SAT expandability, hypoxia, chronic low-grade inflammation, and the release of proinflammatory, diabetogenic, and atherogenic signals [3]. Importantly, adipose tissue (AT) dysfunction can occur even in the absence of generalized obesity, explaining the phenomenon of normal-weight obesity (NWO) and why some lean individuals develop metabolic syndrome. Conversely, some individuals with class III obesity (BMI ≥ 40 kg/m^2^) maintain normal insulin sensitivity, presumably because their SAT expandability remains adequate [15].

### 3.2. Hyperplasia Versus Hypertrophy as Determinants of Expandability

The relative contribution of hyperplasia versus hypertrophy to WAT expansion has profound metabolic implications. Hyperplastic expansion produces a population of small, insulin-sensitive adipocytes that maintain efficient lipid storage and favorable adipokine secretion profiles (high adiponectin, low leptin) [16,17]. These small adipocytes exhibit high capillary density, low inflammatory infiltration, and preserved insulin signaling [18]. However, baseline cell size introduces key complexities; during acute overfeeding, individuals with smaller baseline subcutaneous adipocytes can exhibit worse insulin sensitivity and muscle inflammation. This suggests very small adipocytes may sometimes reflect an exhausted precursor pool unable to recruit new cells, forcing existing adipocytes to undergo rapid, pathological hypertrophy.

In contrast, hypertrophic expansion leads to cellular dysfunction. Hypertrophic adipocytes become hypoxic because the distance for oxygen diffusion exceeds capillary supply; they activate stress pathways including endoplasmic reticulum (ER) stress, mitochondrial dysfunction, and increased reactive oxygen species (ROS) production [19]. These stressed adipocytes secrete pro-inflammatory cytokines such as tumor necrosis factor-alpha (TNF-α), interleukin-6 (IL-6), and monocyte chemoattractant protein-1 (MCP-1), which recruit macrophages and other immune cells into the AT [20]. The resulting inflammatory milieu exacerbates insulin resistance locally and systemically, marking the tipping point where exceeded subcutaneous expandability converts positive energy balance into metabolic dysfunction.

Evidence indicates that hypertrophic expansion of SAT is strongly associated with increased risk of T2D and early metabolic dysfunction, independent of overall adiposity. Lönn et al. [21] conducted a longitudinal, population-based study in a cohort of Swedish middle-aged women, demonstrating that increased subcutaneous abdominal adipocyte size at baseline independently predicted the later development of T2D. This relationship persisted even after accounting for overall body fat and its distribution, underscoring the importance of abdominal adipocyte hypertrophy as a determinant of T2D risk and highlighting the hyperplasia-to-hypertrophy balance as a critical factor in metabolic health. This provides direct clinical validation that physical storage limits (adipocyte size), rather than total fat mass, drive long-term metabolic decline. Complementing these findings, Henninger et al. [22] conducted a case–control study in individuals without obesity with a genetic predisposition to T2D, demonstrating that first-degree relatives exhibited subcutaneous adipocyte hypertrophy along with early metabolic alterations, including reduced insulin sensitivity and elevated triglyceride levels. These changes were accompanied by inflammatory activation, Wnt signaling, and markers of tissue remodeling and fibrosis, supporting the notion that early adipose tissue dysfunction may increase susceptibility to T2D.

### 3.3. Role of Adipose Stem and Progenitor Cells

Maintaining hyperplastic capacity depends fundamentally on the functional integrity of adipose stem cells (ASCs) [23]. These multipotent progenitors reside in the stromal vascular fraction (SVF) of AT and retain the ability to differentiate into mature adipocytes under appropriate physiological cues, as well as into endothelial cells and fibroblastic lineages; however, under chronic pathological stress, their lineage potential can shift toward a pro-fibrotic myofibroblastic phenotype rather than functional adipogenesis, restricting healthy hyperplastic capacity. In healthy individuals, ASCs undergo rounds of proliferation and adipogenic differentiation throughout adult life, enabling AT renewal and expansion [24]. However, several factors can compromise ASC function. With advancing age, ASCs accumulate senescent cells characterized by reduced proliferative and adipogenic capacity; these senescent cells also secrete a senescence-associated secretory phenotype (SASP) that promotes local inflammation and fibrosis [25]. In addition, chronic overnutrition, particularly diets high in fat and sugar, induces oxidative stress and DNA damage in ASCs, thereby impairing their ability to differentiate [26]. Furthermore, genetic polymorphisms in key adipogenic regulators, such as PPARγ and C/EBPα, can influence individual adipose tissue expandability [27]. Supporting this notion, preclinical animal evidence from Zhou et al. [28] highlights the importance of immune–stromal interactions in maintaining ASC functionality within visceral adipose tissue (VAT). In these rodent models, a subset of CX3CR1hi macrophages has been shown to preserve ASC pools during early nutrient excess by limiting cellular senescence and enabling adaptive tissue expansion. In contrast, chronic overnutrition in mice leads to a reduction in these macrophages, accompanied by increased ASC senescence, impaired adipose tissue remodeling, and metabolic dysfunction. Mechanistically, preclinical data reveal that ASCs recruit and modulate these macrophages through MCP-1 signaling and extracellular vesicles, while macrophages counteract senescence via the arginase-1–eIF5A hypusination pathway. Restoration of this macrophage population has been associated with improved ASC maintenance and metabolic outcomes, underscoring the relevance of ASC support systems in obesity-related metabolic deterioration.

### 3.4. Vascularization and Extracellular Matrix as Structural Constraints

Healthy AT expansion requires coordinated angiogenesis (the formation of new blood vessels) and extracellular matrix (ECM) remodeling. As adipocytes enlarge or new adipocytes differentiate, the vascular network must expand to supply oxygen and nutrients and remove metabolic waste [24,29]. Hypoxia—a hallmark of hypertrophic AT—activates hypoxia-inducible factor 1 alpha (HIF-1α), which in turn induces expression of pro-angiogenic factors such as vascular endothelial growth factor (VEGF) [30]. In healthy expansion, this leads to appropriate capillary growth. However, in dysfunctional AT, the angiogenic response is inadequate or dysregulated, resulting in persistent hypoxia and inflammation [31].

SAT has higher angiogenic potential than VAT, which partly explains why SAT expansion is metabolically safer [32]. In individuals with severe obesity, however, SAT angiogenic capacity becomes impaired, contributing to depot-specific dysfunction [33].

The ECM also imposes critical constraints. During AT expansion, the ECM must be remodeled by matrix metalloproteinases (MMPs) and their inhibitors to accommodate new cells [34]. In metabolically challenged states, there is excessive deposition of ECM components, particularly collagen VI, collagen I, and fibronectin. This process, known as AT fibrosis, creates a physical barrier that limits further expansion [35].

### 3.5. Link Between Limited Expandability and Ectopic Lipid Deposition

When the combination of hyperplastic reserve, vascular supply, and ECM pliability reaches its limit, the system fails. Excess dietary lipids can no longer be safely sequestered in SAT and instead accumulate in VAT and ectopic sites (liver, skeletal muscle, pancreas, heart) [36]. Visceral fat generally exhibits a more lipolytic and pro-inflammatory profile than SAT, a biological tendency shaped both by its anatomical location draining directly into the portal circulation and by differences in its cellular and immune composition, including a higher relative density of macrophages and a distinct adipocyte size distribution [37].

**Figure 1:**
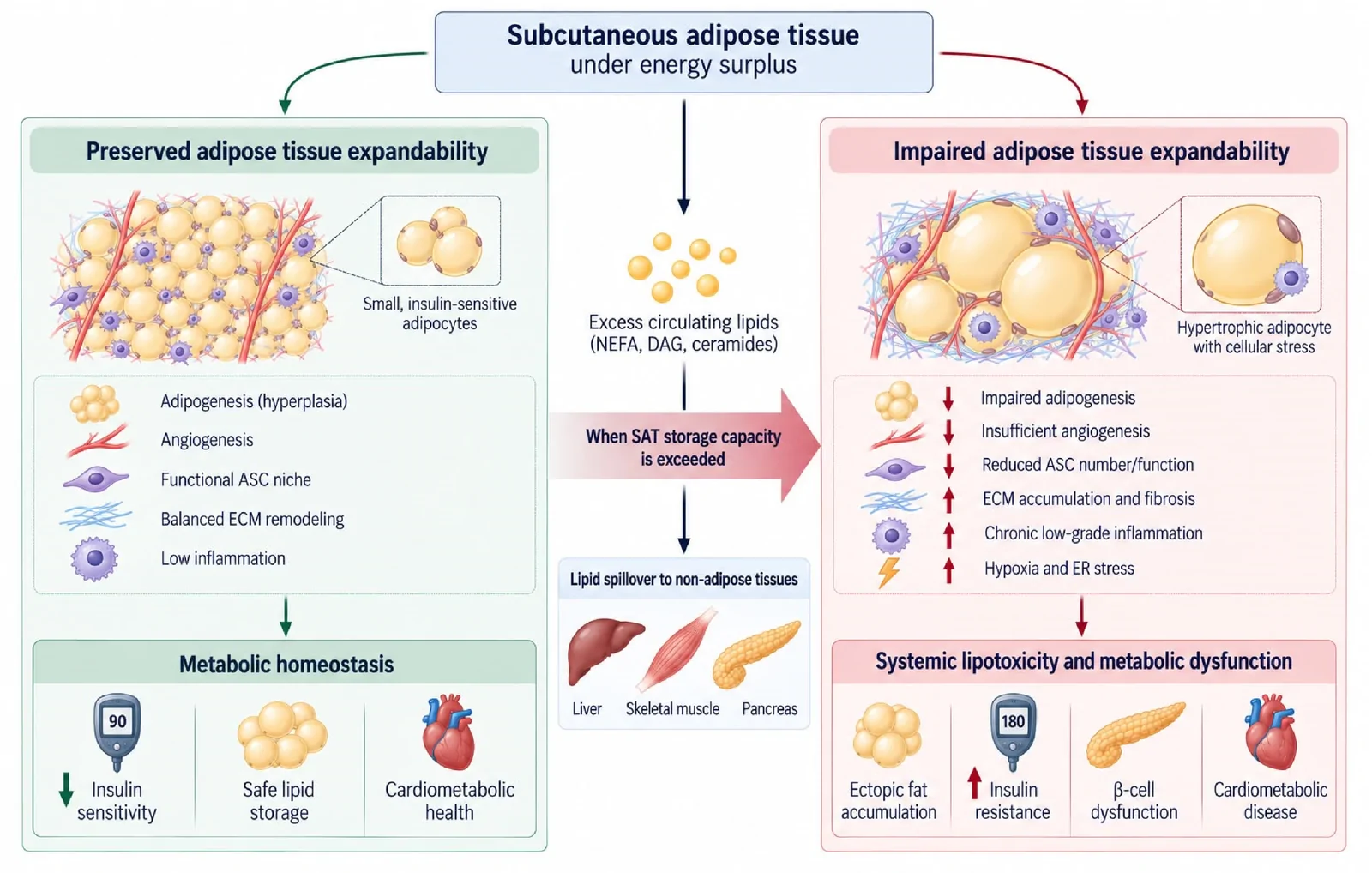
Conceptual model of adipose tissue expandability and metabolic outcomes. Under energy surplus, preserved SAT expandability supports adipogenesis, angiogenesis, and balanced ECM remodeling, maintaining efficient lipid storage and metabolic homeostasis. Conversely, impaired expandability promotes adipocyte hypertrophy, hypoxia, ER stress, inflammation, and fibrosis, ultimately favoring lipid spillover to non-adipose tissues and systemic metabolic dysfunction [7,8,34,38,39,40,42,43]. Abbreviations: ASC, adipose stem cell; DAG, diacylglycerol; ECM, extracellular matrix; ER, endoplasmic reticulum; NEFA, non-esterified fatty acids; SAT, subcutaneous adipose tissue. ↑: increased/upregulation ↓: decreased/downregulation. Created in BioRender. Reytor, C. (2026): BioRender.com.

The concept of lipid spillover explains how limited expandability drives systemic metabolic disease [38]. This progression is summarized in Figure 1.

In the liver, excess free fatty acids (FFAs) drive de novo lipogenesis, leading to metabolic dysfunction-associated steatotic liver disease (MASLD), metabolic dysfunction-associated steatohepatitis, and hepatic insulin resistance [39]. In skeletal muscle, intramyocellular lipid accumulation generates diacylglycerols (DAGs) and ceramides that impair insulin signaling [40]. In the pancreas, lipid deposition (termed non-alcoholic fatty pancreas disease) contributes to β-cell dysfunction and apoptosis [41]. Thus, AT expandability may be conceptualized as a critical physiological buffer for systemic metabolic health. When this buffering capacity is high, metabolic homeostasis is typically preserved; conversely, when expandability is restricted, the threshold for metabolic dysregulation may be reached more readily, irrespective of total adiposity.

## 4. Metabolic Consequences of Impaired Expandability

### 4.1. Adipocyte Hypertrophy, Cellular Stress, and Lipotoxic Signaling

Once adipocytes exceed a critical size threshold, a cascade of cellular stress responses is activated. The mechanical stretch of the plasma membrane triggers the unfolded protein response due to ER overload. The UPR involves activation of three canonical sensors: PERK, IRE1α, and ATF6. While transient UPR activation is adaptive, chronic UPR activation in hypertrophic adipocytes leads to apoptosis via CHOP and JNK pathways, representing a cellular “point of no return” that depletes the functional storage pool and accelerates lipid spillover [44].

In vitro and animal models demonstrate that mitochondria in hypertrophic adipocytes become fragmented and dysfunctional [45]. Consistent with this, González et al. [46] conducted a multi-omics study in diet-induced obese mice to assess metabolic adaptability following combined dietary and exercise interventions. Although systemic phenotypes improved, VAT continued to exhibit marked mitochondrial abnormalities, including disrupted structure and deficits in metabolic and proteostatic functions. These findings suggest that obesity leads to lasting mitochondrial impairments that are not completely reversible, thereby constraining the metabolic flexibility of adipose tissue. This dysfunction results in decreased fatty acid oxidation, increased ROS production, and accumulation of lipid intermediates such as ceramides and DAGs [42], which directly impair insulin signaling by activating protein kinase C (PKC) isoforms (particularly PKCθ in muscle and PKCε in liver) and by promoting serine phosphorylation of insulin receptor substrate (IRS) proteins [43,47]. These pathways suggest that surpassing subcutaneous expandability thresholds inflicts organelle-level damage that cannot be fully corrected by caloric restriction alone.

Importantly, the relationship between adipocyte size and metabolic dysfunction is non-linear. The “critical size threshold” posits that once adipocytes surpass a certain diameter, they become irreversibly dysfunctional [48]. In accordance with this concept, Cotillard et al. [49] conducted a study in two cohorts of women with severe obesity undergoing bariatric surgery, identifying adipocyte volume thresholds above which the risk of T2D was significantly increased and metabolic improvement after surgery was reduced. This threshold varies among individuals and depots, but accumulating evidence suggests that larger adipocytes are consistently associated with greater insulin resistance, higher inflammatory markers, and increased T2D risk [50].

### 4.2. Chronic Inflammation and Immune Cell Infiltration

Dysfunctional AT is not merely a passive storage organ but an active driver of systemic low-grade inflammation [51]. The inflammatory cascade begins with hypertrophic adipocytes secreting chemokines (e.g., MCP-1, RANTES) that recruit circulating monocytes into the AT [52]. Once recruited, monocytes differentiate into macrophages and, under the influence of local signals, exhibit diverse activation states. While conventionally described using the simplified in vitro M1 (pro-inflammatory) and M2 (anti-inflammatory) polarization paradigm, in vivo adipose tissue macrophages exist on a highly complex, continuous spectrum of activation phenotypes [53]. Stressed adipocytes stimulate M1 macrophages to secrete TNF-α, IL-1β, IL-6, and inducible nitric oxide synthase [54]. These cytokines act in a paracrine manner to exacerbate adipocyte insulin resistance and in an endocrine manner to promote hepatic and muscle insulin resistance [55]. In fact, in dysfunctional AT, the cellular balance shifts toward a highly pro-inflammatory tissue microenvironment, driven by the loss of regulatory T cells and protective, tissue-resident macrophage populations: adipose tissue macrophages can constitute up to 40–50% of the SVF in obese AT, compared with 10–15% in lean AT [56].

Beyond macrophages, other immune cell types infiltrate dysfunctional AT, contributing to its pro-inflammatory environment. Preclinical animal models have mapped these specific inflammatory dynamics, demonstrating that CD8+ T cells promote inflammation and facilitate adipose tissue macrophage recruitment [57], while B cells produce pathogenic antibodies and secrete pro-inflammatory cytokines such as IL-6 [52]. Mast cells further exacerbate tissue remodeling by releasing chymase and tryptase, thereby contributing to adipose tissue fibrosis [58]. Moreover, neutrophils intensify the inflammatory response by releasing neutrophil extracellular traps [59]. In contrast, protective immune populations, including regulatory T cells and M2 (anti-inflammatory) macrophages, are reduced in dysfunctional AT, shifting the balance toward sustained inflammation. This chronic inflammatory state has systemic consequences, as elevated circulating levels of IL-6 and TNF-α impair insulin signaling in the liver and skeletal muscle [51]. Concurrently, increased C-reactive protein (CRP) levels heighten cardiovascular risk, while inflammatory cytokines stimulate hepatic gluconeogenesis and lipogenesis, thereby exacerbating hyperglycemia and dyslipidemia [60,61].

### 4.3. Fibrosis as a Mechanical and Metabolic Barrier

AT fibrosis arises both as a consequence of chronic overnutrition and as a driver of impaired tissue expandability [26]. This process is mediated by the activation of adipose tissue fibroblasts, which originate from the same ASC pool that typically differentiates into adipocytes. Under conditions of chronic hypoxia, mechanical stress, and persistent inflammatory signaling—particularly through TGF-β1—these fibroblasts differentiate into myofibroblasts that deposit excessive ECM components [62]. As a result, key ECM proteins become upregulated, including collagens I, III, and VI, which are the most abundant and whose cross-linking increases tissue stiffness; fibronectin, which promotes cell adhesion and migration; and lysyl oxidase (LOX), which facilitates collagen cross-linking and enhances tensile strength [63]. The accumulation of these components creates a mechanical barrier that restricts adipocyte expansion and disrupts vascular ingrowth [62]. This physical constraint may modulate the “personal fat threshold” framework, suggesting that tissue stiffness can act as an active, non-genetic bottleneck that limits safe subcutaneous expansion. Moreover, the fibrotic ECM acts as a scaffold that sustains inflammation by retaining immune cells within the tissue. In humans, SAT fibrosis has been strongly associated with hepatic insulin resistance and MASLD [64]. Yang et al. [65] conducted a cross-sectional analysis of 904 patients with MASLD from the NHANES 2017–2020 cohort and demonstrated that insulin resistance-related indices were significantly associated with liver fibrosis (LF), with TyG-WHtR emerging as an independent predictor (OR = 2.69; 95% CI: 2.08–3.47). Notably, a multivariable predictive model incorporating IR markers and clinical parameters achieved good discriminative performance, highlighting the relevance of metabolic dysfunction in fibrosis progression and reinforcing the interplay between adipose tissue dysfunction, insulin resistance, and hepatic fibrogenesis.

Notably, fibrosis is not uniformly present in all individuals with obesity; those classified as MHO exhibit minimal fibrosis, whereas individuals with MUO display pronounced ECM accumulation [2]. This distinction suggests that fibrosis represents a critical structural determinant in the progression from benign to pathogenic obesity, although the rate of development and potential for resolution exhibit substantial inter-individual heterogeneity [66]. Consequently, therapeutic strategies targeting AT fibrosis, such as LOX inhibitors and MMP activators, are currently being explored as potential interventions for obesity-related metabolic disorders [62].

### 4.4. Lipid Spillover to Liver, Muscle, and Pancreas

When SAT expandability is exhausted and fibrosis prevents further expansion, dietary and endogenously synthesized lipids are redirected to ectopic storage sites. Each site has distinct pathophysiological consequences. In the liver, hepatic lipid accumulation is among the earliest consequences of expandability failure. Excess FFAs enter hepatocytes via CD36 and fatty acid transport proteins [67]. Hepatic DAG accumulation activates PKCε, which impairs insulin receptor signaling by phosphorylating and inhibiting the insulin receptor [68]. This leads to increased gluconeogenesis (via FOXO1 activation) and very-low-density lipoprotein (VLDL) secretion, contributing to hyperglycemia and hypertriglyceridemia [68]. MASLD can progress to MASH, with fibrosis, inflammation, and increased risk of cirrhosis and hepatocellular carcinoma [69].

In skeletal muscle, intramyocellular lipids accumulate when FFA availability surpasses the tissue’s oxidative capacity. DAG accumulation activates PKCθ, which inhibits insulin signaling, reducing GLUT4 translocation and glucose uptake [70]. This muscle insulin resistance is a major determinant of whole-body glucose intolerance. Interestingly, endurance-trained athletes also have high intramyocellular lipid but maintain insulin sensitivity (“athlete’s paradox”) because their lipid droplets are small, dynamic, and associated with high oxidative capacity—highlighting that lipid composition and droplet dynamics matter as much as total lipid content [71]. Pancreatic fat accumulation, characteristic of non-alcoholic fatty pancreas disease, impairs β-cell function through lipotoxic mechanisms. In particular, the accumulation of toxic lipid intermediates such as ceramides promotes β-cell apoptosis, ultimately reducing insulin secretory capacity [72]. As β-cell function declines, this impairment may drive the transition from compensated insulin resistance, where normoglycaemia is maintained, to decompensated hyperglycaemia characteristic of T2D. Consistent with this pathophysiological framework, pancreatic fat content has been reported to be inversely associated with insulin secretion [73]. Excess lipid deposition in the heart contributes to lipotoxic cardiomyopathy, which is marked by diastolic dysfunction and reduced contractile performance, and increased risk of heart failure with preserved ejection fraction—a common phenotype in obesity [74].

### 4.5. Associations with Insulin Resistance, Type 2 Diabetes, and Cardiometabolic Risk

The clinical correlate of impaired expandability is a spectrum of metabolic disorders. The hypertriglyceridemic waist phenotype—defined by increased waist circumference and elevated triglyceride levels—is a clinical marker of impaired SAT expandability. Individuals with this phenotype have disproportionately small, dysfunctional SAT and high VAT, with greatly increased risk of T2D and cardiovascular disease [75,76].

Building on this clinical framework, population-based imaging studies provide further insight into the metabolic role of adipose tissue distribution. The Framingham Heart Study reported that abdominal SAT is not associated with a linear increase in cardiometabolic risk, and among individuals with high visceral adiposity, greater SAT was associated with lower triglyceride levels and a lack of worsening of other metabolic abnormalities [77]. These findings suggest that SAT may exert a protective or buffering role in the context of excess lipid burden. Supporting this concept at the cellular level, longitudinal studies have demonstrated that adipocyte hypertrophy—reflecting limited storage capacity—is a strong predictor of incident T2D independently of BMI, suggesting that the metabolic impact of SAT depends not only on its quantity but also on its capacity for healthy expansion [78,79].

## 5. Dietary Determinants of Adipose Expandability

### 5.1. Energy Surplus and Trajectory of Adipose Expansion During Weight Gain

The process of WAT expansion begins with chronic energy surplus, but the trajectory and metabolic consequences of expansion vary dramatically among individuals. A key insight from overfeeding studies is that the rate of weight gain and the composition of excess energy influence expandability outcomes [80].

In a prospective overfeeding study, Johannsen et al. [81] fed 29 healthy men 40% above their baseline energy requirements for 8 weeks, resulting in a mean weight gain of 7.6 kg (55% fat). Despite similar weight gain, there was marked heterogeneity in metabolic responses. Insulin sensitivity decreased by 18% overall, and intrahepatic lipid increased by a mean of 46%. Counter to the traditional “adipose tissue expandability” hypothesis, individuals with smaller subcutaneous adipocytes at baseline had a greater decrease in insulin sensitivity and greater upregulation of inflammatory genes in skeletal muscle. In contrast, larger adipocytes did not promote ectopic lipid accumulation. The authors concluded that smaller fat cells predict a worsened metabolic response to overfeeding but did not identify the underlying mechanism. This finding challenges a “simple” expandability model where small cells always equal health, perhaps suggesting that very small cells in some contexts represent a depleted precursor pool rather than high expansion capacity. As reviewed by Vishvanath & Gupta [82], when ASCs become exhausted or fail to differentiate, neoadipogenesis is impaired. In this context, even modest overfeeding forces existing small adipocytes to hypertrophy rapidly, leading to cellular stress, inflammation, and insulin resistance.

The concept of personal fat threshold posits that each individual has a maximum amount of fat that can be safely stored in SAT. Taylor et al. [83] directly tested the personal fat threshold hypothesis in 20 individuals with T2D who had a mean BMI of only 24.8 kg/m^2^. Participants underwent a structured weight loss program of up to three cycles of a low-calorie diet (600–800 kcal/day), followed by weight stabilization. The key findings were that a median weight loss of just 6.5% was sufficient to achieve T2D remission in 70% of participants. This metabolic improvement was associated with a significant 46% reduction in intrahepatic lipid, a marked decrease in markers of hepatic de novo lipogenesis, and restoration of insulin sensitivity and beta-cell function toward levels seen in normoglycemic controls. Notably, while weight loss reduced visceral and subcutaneous fat, post-weight loss VAT remained significantly higher in women with T2D compared to BMI-matched controls, suggesting that the ability to safely store fat in SAT may be a key determinant of metabolic health. These data support the personal fat threshold hypothesis by demonstrating that individuals with a low threshold develop T2D at a relatively low BMI and that this diabetes can be reversed by reducing fat stores below their personal threshold.

### 5.2. Macronutrient Composition Shaping Expandability

#### 5.2.1. Preclinical and Mechanistic Insights

The type of dietary fat consumed during weight gain profoundly affects AT expandability. Saturated fatty acids (SFAs; e.g., palmitic acid, stearic acid) are particularly detrimental because they (1) induce ER stress and inflammation in adipocytes directly, (2) promote ceramide synthesis, and (3) impair adipocyte differentiation [84].

In contrast, polyunsaturated fatty acids (PUFAs; particularly omega-3 EPA and DHA) support healthy expansion [85]. Mechanistically, ω-3 PUFAs activate ciliary FFAR4 signaling to drive adipogenesis, enabling the formation of new adipocytes to store excess lipids without triggering hypertrophy-induced dysfunction [86]. Similarly, dietary MUFAs promote healthy white adipose tissue expansion, reduce inflammatory macrophage infiltration, and improve metabolic syndrome outcomes [87]. Despite these neutral findings in some human overfeeding studies, omega-3 PUFAs exert additional benefits at the molecular level: they activate PPARγ (the master regulator of adipogenesis), promote angiogenesis, reduce inflammation, and enhance mitochondrial biogenesis [88].

At the endocrine level, high insulin levels directly inhibit lipolysis, trapping FFAs in AT [89]. In the context of limited expandability, this is paradoxical: insulin-induced trapping might seem beneficial, but it prevents the mobilization of FFAs for oxidation in other tissues, potentially exacerbating adipocyte hypertrophy [90]. Chronically high insulin levels also downregulate insulin receptors on adipocytes, promoting resistance and further hyperinsulinemia [91]. Unlike glucose, fructose has unique metabolic effects; it is metabolized primarily in the liver, where it drives de novo lipogenesis, increases hepatic DAG and ceramide, and promotes MASLD independent of SAT expansion [92]. Fructose also impairs satiety signaling, promoting overconsumption [93]. Consequently, excessive fructose consumption is hypothesized to contribute to features of the “expandability failure” phenotype, with high VAT and high intrahepatic lipid despite relatively modest SAT expansion [94].

Regarding amino acid signaling, dietary protein influences ASC function through multiple mechanisms. Amino acids—particularly leucine, arginine, and glutamine—activate the mTOR signaling pathway, which regulates cell growth and differentiation [95]. Moderate protein intake supports ASC proliferation and adipogenesis, whereas protein restriction impairs hyperplastic capacity [96]. In an animal model, long-term (60–120 days) intake of a high-protein diet improved insulin sensitivity, increased glucose uptake and lipogenesis in adipocytes, and modulated endocrine function (decreased leptin secretion), whereas a high-fat diet had inverse negative effects, including reduced adiponectin secretion, increased leptin and resistin, and diminished lipogenesis [97]. However, excessive protein intake may have adverse effects by increasing IGF-1 signaling, which can promote adipocyte hypertrophy rather than hyperplasia [98].

#### 5.2.2. Human Clinical and Intervention Evidence

To translate these cellular pathways into clinical outcomes, overfeeding trials and cohort studies provide essential insights. In a randomized controlled trial, Rosqvist et al. [99] reported that overfeeding with SFAs or PUFAs in individuals with overweight resulted in comparable weight gain and did not differentially affect lean tissue accumulation, with no clear differences in circulating metabolic regulators. These findings suggest that, in the context of short-term caloric excess, the type of dietary fat may not substantially influence tissue composition, highlighting a potential discrepancy between mechanistic insights and outcomes observed in human intervention studies. Nevertheless, human clinical trials indicate that specific lipid interventions may influence metabolic outcomes; for example, fish oil supplementation has been shown to improve insulin sensitivity [100].

With respect to carbohydrates, the carbohydrate–insulin model proposes that high-glycemic carbohydrates increase insulin secretion, which in turn shifts nutrient partitioning toward fat storage in AT, increases hunger, and promotes positive energy balance [101]. While the carbohydrate–insulin model remains debated, there is strong evidence that carbohydrate quality modulates expandability [102]. High-glycemic index carbohydrates cause rapid glucose absorption, high insulin spikes, and subsequent hypoglycemia, which promotes further eating and favors lipid storage. In contrast, low-glycemic index carbohydrates produce gradual glucose absorption, lower insulin responses, and support metabolic flexibility [103].

Finally, the translation of dietary protein requirements is closely linked to human behavior; early-life protein intake is particularly important. The protein leverage hypothesis suggests that humans and animals regulate protein intake more strongly than carbohydrate or fat intake [104]. When dietary protein is diluted by processed carbohydrates and fats, individuals overconsume total calories to reach their protein target, promoting obesity. This may partly explain the obesity epidemic in populations consuming highly processed, low-protein-content diets [105].

### 5.3. Micronutrients and Bioactive Compounds

At the preclinical and molecular level, several micronutrients and phytochemicals play a critical role in regulating adipose tissue expandability. Cellular models show that activation of the vitamin D receptor promotes adipose stem cell differentiation and inhibits fibrosis [106]. Zinc is also essential, as it is required for proper PPARγ function [107], and its deficiency can impair adipocyte differentiation [108]. In addition, various polyphenols, including resveratrol, epigallocatechin gallate, and curcumin, contribute to improved expandability by activating AMPK, inhibiting TGF-β signaling, reducing fibrosis, and promoting angiogenesis [109,110,111,112]. Beyond these factors, the gut microbiota plays a significant role by producing short-chain fatty acids (SCFAs), such as acetate, propionate, and butyrate, which regulate adipogenesis, inflammation, and overall energy homeostasis [113,114,115]. However, diet-induced dysbiosis, characterized by low fiber and high fat intake, can reduce SCFA production and contribute to adipose tissue dysfunction [116]. In translational and human clinical settings, micronutrient status correlates strongly with adipose health and metabolic syndrome risk. Observational studies show that vitamin D deficiency, which is common in obesity, is associated with impaired adipogenesis and increased inflammation [117,118,119]. Similarly, low magnesium levels have been linked to insulin resistance and heightened inflammatory responses, as reported in a meta-analysis by Mashayekhi et al. [120]. Importantly, the clinical relevance of micronutrient status extends beyond mechanistic regulation of adipose tissue biology, particularly in the context of bariatric surgery, where deficiencies are highly prevalent. Specifically, Schiavo et al. [121] conducted a retrospective study of 80 patients undergoing sleeve gastrectomy and demonstrated that individuals who received preoperative correction of micronutrient deficiencies did not develop new deficiencies at 3- and 12-month follow-up, whereas those without prior correction remained deficient despite standardized postoperative supplementation. These findings highlight the importance of adequate micronutrient status not only for metabolic function but also for preventing persistent deficiencies in high-risk populations.

### 5.4. Dietary Patterns and Depot-Specific Responses

The Western diet, characterized by a high intake of SFAs, refined grains, sugars, and low fiber content, is a major contributor to both obesity and chronic low-grade inflammation [122]. Typically composed of fast and processed foods, sweets, high-fat products, and sugar-sweetened beverages, this dietary pattern promotes an “unhealthy expandability” phenotype in adipose tissue, and therefore metabolic dysfunction [123]. The Mediterranean diet, characterized by a high content of protective compounds including MUFAs, PUFAs, fiber, and polyphenols, supports healthy adipose tissue expansion and has been shown to reduce VAT and intrahepatic lipid content even in the absence of weight loss, while also improving immune and inflammatory responses, partly through the reduction in MASLD [124,125].

Depot-specific responses are important: SAT is more responsive to dietary interventions than VAT. For example, weight loss diets reduce SAT volume more than VAT volume [126]. This concept is supported by a comprehensive systematic review and meta-analysis by Merlotti et al. [127], which included 89 trials and found that, at baseline, subcutaneous fat area, volume, and weight were significantly greater than the corresponding visceral fat measures. Furthermore, the absolute reduction in subcutaneous fat was significantly greater than that of visceral fat. However, because SAT has a much larger baseline volume than VAT, the same absolute reduction represents a smaller percentage of the SAT depot. Consequently, when expressed as a percentage change, the decrease in visceral fat was consistently greater than that of subcutaneous fat across all intervention types (diet, drugs, surgery), with no single strategy preferentially targeting the visceral depot. Visceral fat loss was strongly correlated with subcutaneous fat loss, suggesting that overall fat reduction is a key driver of improvements in both depots.

### 5.5. Distinction Between Expandability During Weight Gain Versus Weight Loss

A critical but often overlooked point is that expandability during weight gain and during weight loss are not symmetric. Weight loss reduces lipid burden, relieves mechanical stress, and improves AT function, but it does not necessarily restore intrinsic expandability (hyperplastic capacity, ASC function, reversal of fibrosis) [128]. Many individuals who lose substantial weight remain at risk for metabolic deterioration upon regain because their underlying expandability deficit persists [82,129].

This has profound clinical implications: interventions aimed at preserving expandability might yield greater benefit if explored early in the weight gain trajectory, before fibrosis and ASC exhaustion become irreversible [35,130]. Once established, AT fibrosis may present a highly persistent structural constraint that is resistant to standard weight-loss interventions, though post-surgical remodeling suggests some degree of tissue plasticity is possible. In the bariatric surgery context, as discussed below, the dramatic benefits derive primarily from reducing lipid overflow, not from reversing structural expandability limitations [131,132].

## 6. Bariatric Surgery as a Natural Experiment in Adipose Expandability

### 6.1. Bariatric Surgery as a Metabolic Intervention That Reduces Lipid Overflow Rather than Restoring Expandability

Bariatric surgery, including Roux-en-Y gastric bypass and sleeve gastrectomy, is the most effective treatment for severe obesity, producing weight loss, resolution of T2D, and dramatic reductions in cardiovascular risk [133,134]. However, the mechanisms underlying these benefits remain incompletely understood. Available evidence suggests that reduction in lipid overflow may be one major mechanism of metabolic improvement after bariatric surgery, while the degree to which intrinsic adipose-tissue expandability is restored remains uncertain [135]. It is important to note that this adipose-centric perspective is an interpretive model rather than an exclusive mechanism. Bariatric surgery also leverages parallel, weight-independent pathways, including post-surgical surges in gut hormones, ghrelin suppression, altered bile acid signaling, and gut microbiota reorganization, which collectively support systemic metabolic recovery.

De Souza et al. [136] conducted a prospective cohort study including 23 patients with class II–III obesity undergoing Roux-en-Y bariatric surgery that were evaluated to assess changes in inflammatory, biochemical, and oxidative stress parameters before and three months after the intervention. Paired analyses revealed significant reductions in BMI, total cholesterol, triglycerides, low-density lipoprotein, high-density lipoprotein (HDL), CRP, and reactive oxygen species production, as measured by dichlorofluorescein. Additionally, antioxidant status improved, as evidenced by increased superoxide dismutase activity, although levels of reduced glutathione decreased. Importantly, while several studies report increases in HDL post-surgery [137,138,139,140], De Souza et al. [136] attribute the early HDL reduction mainly to a concurrent reduction in total cholesterol and the physiological kinetics of rapid weight loss during the first six postoperative months. Importantly, this acute drop in HDL does not necessarily indicate metabolic worsening, but rather represents a structural and functional reorganization of HDL subspecies. This process is characterized by a qualitative transition from ApoE-containing particles to more functional, ApoA1-rich phenotypes with superior antioxidant capacity. Additionally, temporary post-surgical sedentary behavior might also play a role in this transient decline. Together, these findings indicate that bariatric surgery not only improves lipid and anthropometric profiles but also attenuates systemic inflammation and oxidative stress, effects that are consistent with reduced ectopic lipid burden and improved metabolic homeostasis.

After BS, dramatic reductions in caloric intake (and, after Roux-en-Y gastric bypass [RYGB], malabsorption) lead to rapid mobilization of lipid stores. Within weeks, SAT and VAT volumes decrease, hepatic steatosis resolves, and pancreatic fat content declines [133]. This reduction in ectopic lipid accumulation relieves the lipotoxic burden on liver, muscle, and pancreas, improving insulin sensitivity and β-cell function, and reducing cardiometabolic risk [38,141]. Notably, these metabolic improvements occur before substantial weight loss, supporting the overflow reduction hypothesis [142].

However, evidence regarding the extent to which BS reverses established AT fibrosis or restores hyperplastic capacity remains mixed; fibrotic ECM is remarkably stable since collagen cross-links have half-lives of months to years; ASCs from post-BS patients often remain senescent, with impaired adipogenic potential [7,143,144]. Thus, the fundamental expandability deficit may persist, leaving patients vulnerable to metabolic deterioration if weight regain occurs [132]. However, despite this persistent structural vulnerability, some functional improvements in adipose tissue appear to be sustained even after weight regain. Kerr et al. [145] conducted a five-year transcriptomic study of women following RYGB surgery showing that inflammatory gene expression in subcutaneous white adipose tissue continued to decrease between years 2 and 5—a period of average 8 kg weight regain—and 71 of these genes correlated with improved adipocyte morphology and serum adipokine levels. This suggests that while fibrotic expandability deficits may persist, the anti-inflammatory remodeling of adipose tissue can be weight-regain-independent and may contribute to the long-term metabolic protection observed after bariatric surgery.

### 6.2. Differential Resolution of Ectopic Fat Versus Intrinsic Adipose Storage Capacity

BS effectively reverses ectopic lipid deposition, but the effects on intrinsic SAT structure and function are more complex. Consistent with this, Adnan et al. [146] conducted a longitudinal study comparing ASCs before and after weight loss which demonstrated significant improvements in adipose tissue phenotype, including reduced adipocyte size, decreased circulating leptin, and increased adiponectin levels, together with the disappearance of crown-like structures, indicating attenuated inflammation. At the cellular level, ASCs exhibited enhanced mitochondrial respiration and proliferative capacity, along with downregulation of pro-inflammatory markers. However, their immunomodulatory behavior remained context-dependent in coculture with macrophages, displaying both pro- and anti-inflammatory responses, suggesting that weight loss induces substantial metabolic and inflammatory improvements, but does not fully normalize stromal cell function.

Nonetheless, these improvements may not be uniformly sustained in all patients. Weight regain and the partial loss of metabolic benefits have been associated with persistent adipose tissue dysfunction, in which fibrosis is increasingly recognized as a central pathological mechanism limiting tissue remodeling, impairing metabolic flexibility, and influencing long-term prognosis. Recent advances in adipose tissue biology further highlight that fibrosis is not merely a consequence of inflammation, but a structural constraint that restricts adipose expandability and may contribute to long-term metabolic vulnerability despite initial post-surgical improvement [147].

A key finding is that pre-BS AT fibrosis predicts poorer metabolic outcomes after surgery. Individuals with high SAT collagen content have lower T2D remission rates and greater risk of weight regain [147]. This suggests that fibrosis is not merely a marker of obesity severity but an independent determinant of surgical response. Interventions targeting AT fibrosis (i.e., LOX inhibitors, exercise) could potentially enhance BS outcomes [148,149,150,151].

These findings—specifically the sustained reduction in inflammatory gene expression despite weight regain and the post-surgical improvements in mitochondrial respiration and progenitor-cell proliferation—demonstrate that lipid overflow reduction and active tissue remodeling are not mutually exclusive, but rather operate as complementary, synergistic arms of metabolic recovery. In this integrated cascade, the rapid alleviation of systemic nutrient pressure (overflow reduction) serves as a vital permissive trigger. By immediately relieving severe mechanical, hypoxic, and inflammatory stress from the adipocytes, this systemic off-loading provides the necessary physiological environment for the tissue to initiate long-term biological remodeling, even if the absolute physical limits of expansion remain ultimately constrained by persistent factors such as collagen cross-linking.

### 6.3. Pre-Surgical Expandability as a Determinant of Metabolic Response and Diabetes Remission

Heterogeneity in BS outcomes can be partially explained by pre-surgical expandability. In a large prospective cohort study (*n* = 161), Ledoux et al. [152] characterized SAT and VAT from patients with obesity undergoing BS. Prior to surgery, insulin-resistant patients exhibited SAT enriched in adipogenic progenitors and CD4+ T lymphocytes, along with VAT adipocyte hypertrophy and increased macrophage infiltration. These tissue-specific alterations were associated with poorer weight loss and reduced improvements in insulin sensitivity at 1 and 3 years post-BS.

Additionally, myofibrogenic progenitor populations were identified as common determinants of post-surgical weight and metabolic outcomes. Their abundance was associated with less favorable trajectories of excess weight loss and insulin resistance. Overall, the findings suggest that SAT and VAT cellular composition contributes to both metabolic dysfunction in obesity and variability in response to bariatric surgery, highlighting AT remodeling as a potential determinant of individual outcomes.

### 6.4. Implications for Understanding Weight Regain and Heterogeneity in Long-Term Outcomes

Weight regain after BS is common, as demonstrated in a systematic review and meta-analysis by Reis et al. [153], which found that 49% of patients experienced weight regain after bariatric surgery, with rates of 42% after Roux-en-Y gastric bypass and up to 64% in European cohorts, highlighting that long-term weight maintenance requires ongoing lifestyle and dietary interventions beyond surgery [154]. The expandability framework provides a mechanistic explanation: if BS reduces lipid overflow but does not fully restore intrinsic expandability, then when patients slowly return to positive energy balance (due to dietary non-adherence, poor dietary compliance, hormonal adaptation, or lifestyle factors) [155,156], the same structural limitations that existed pre-surgery will constrain safe lipid storage. Lipid overflow will recur, and metabolic dysfunction will reappear [132].

This framework offers a plausible explanation for the metabolic heterogeneity observed during weight regain. Specifically, individuals with preserved expandability may safely replenish subcutaneous stores without immediate metabolic decline, whereas those with restricted expandability may undergo rapid lipid spillover, triggering recurrent MASLD and metabolic dysfunction even with minor weight regain. The post-BS metabolic phenotype is thus determined largely by the interaction between the degree of caloric restriction maintenance and the underlying expandability reserve [157].

Long-term management of BS patients should therefore focus on strategies that protect expandability: continued dietary quality (low SFA, high MUFA/PUFA, low glycemic load), regular exercise (which promotes angiogenesis and reduces fibrosis), and weight stability [31,158,159,160]. Pharmacological agents that improve AT function (e.g., GLP-1 receptor agonists, thiazolidinediones) may also have a role in selected patients [161].

## 7. Clinical Implications, Knowledge Gaps, and Future Directions

The expandability framework is a research model that redefines obesity-related metabolic risk beyond the traditional reliance on BMI and waist circumference [162]. The ability of WAT to expand in a healthy manner is a key feature of metabolic homeostasis, as it limits pathological adipocyte hypertrophy and prevents ectopic lipid deposition in organs such as the liver and skeletal muscle [9]. Although this model requires further clinical validation, we propose a hypothetical, three-tiered risk-stratification framework to conceptualize how metabolic risk scales with tissue-level dysfunction: High Expandability (MHO phenotype): Individuals have a low risk of T2D and cardiovascular disease, where the primary goal is to preserve expandability through healthy lifestyle modifications [4]; Intermediate Expandability (Early MUO with minimal fibrosis): This group is hypothesized to be at elevated risk of progression to established disease and could benefit from targeted lifestyle strategies aimed at mitigating early fibrogenesis [163]; Low Expandability (Established MUO with fibrosis and ectopic lipid deposition): These individuals may represent a high-risk metabolic phenotype in whom future translational approaches might explore combining weight reduction (through diet, pharmacotherapy, or bariatric surgery) combined with adjunctive anti-fibrotic strategies [164,165]. Such a framework would function as a roadmap for future research and allow for personalized prevention and the allocation of resources to those most likely to benefit [166].

Evaluating this expandability-based framework in future clinical research settings could help operationalize personalized approaches to cardiovascular disease (CVD) risk assessment. By shifting the clinical focus from static anthropometric measures to the dynamic functional capacity of adipose tissue, this model provides an early indicator for metabolic decompensation [164]. Specifically, it allows clinicians to identify individuals with “intermediate expandability”—who are normoglycemic but already showing early fibrotic signs—before they develop overt dyslipidemia, hypertension, or hyperglycemia. Early identification enables timely, targeted lifestyle interventions that directly mitigate the key pathways of CVD progression: systemic low-grade inflammation and atherogenic dyslipidemia driven by ectopic lipid overflow. Consequently, investigating this framework within primary care research models may ultimately offer insights into early CVD risk stratification by focusing on adipose tissue function, rather than waiting for traditional cardiovascular risk factors to manifest and require pharmacological management.

Given that advanced fibrosis and ASC exhaustion may be highly resistant to reversal once established, strategies aimed at preserving expandability might yield greater efficacy when explored early in the weight gain trajectory; conceptually, a potential intervention window before reaching obesity (according to BMI) is proposed for investigation [167,168]. Although this threshold is not a rigid clinical diagnostic cutoff, it is a conceptual boundary based on preclinical evidence showing that the aforementioned pathways can be dramatically accelerated as individuals transition into established, severe obesity. Key nutritional strategies to maintain expandability are shown in Table 1. Any dietary measure should be combined with regular physical activity (≥150 min/week of moderate-to-vigorous exercise) to promote angiogenesis and reduce adipose tissue inflammation [164,169].

The validation and clinical translation of non-invasive or minimally invasive biomarkers of expandability represent an urgent priority. Candidate biomarkers include SAT biopsy-derived adipocyte size distribution (with a high ratio of small-to-large adipocytes correlating with insulin sensitivity) [183,184], circulating markers of fibrosis, such as PRO-C3 (N-terminal propeptide of collagen III), which has been shown to be associated with advanced liver fibrosis and improves after bariatric surgery [185]; adipokine profiles including high adiponectin and low leptin (adjusted for fat mass); inflammatory markers such as low high-sensitivity CRP, IL-6, and TNF-α [186]; metabolomic and lipidomic panels, as ceramide ratios have been strongly implicated in adipocyte dysfunction underlying metabolic diseases [187]; gene expression in blood, where Mest expression has been proposed as an early biomarker of adipose tissue expansion [188]; and advanced imaging techniques (magnetic resonance imaging-proton density fat fraction for liver fat, computed tomography for the visceral-to-subcutaneous adipose tissue ratio) to assess ectopic lipid deposition, which indirectly reflects expandability failure, and proton magnetic resonance spectroscopy for intramyocellular lipids [189].

Despite the theoretical appeal of the expandability hypothesis, major knowledge gaps remain. There is a notable lack of longitudinal studies directly assessing adipose tissue expansion capacity over years or decades, as most evidence is cross-sectional or derived from short-term overfeeding studies. Our understanding of ASC dynamics in humans is limited; although human ASCs have been investigated for correcting obesity-induced metabolic dysregulation, larger and more rigorous validation studies are needed [190]. No validated non-invasive measure of adipose tissue fibrosis is currently established for routine clinical practice; although magnetic resonance elastography (MRE) and diffusion-weighted imaging (DWI) show promise, their application to the routine assessment of AT fibrosis remains investigational [191,192]. Sex- and age-specific differences in expandability are poorly characterized. Women generally have greater expandability than men, but the underlying mechanisms (e.g., estrogen effects on ASCs, different depot distribution) remain incompletely understood. Furthermore, expandability declines with age, but the trajectory and modifiability of this decline are largely unknown [193]. Gene–environment interactions that determine expandability are largely unexplored; however, genome-wide association studies have identified loci near PPARG, FTO, and MC4R that influence obesity, and recent work has identified 62 loci where the same allele is associated with both higher adiposity and lower cardiometabolic risk, with genes prioritized supporting key roles in fat distribution (FAM13A, IRS1, and PPARG) and adipocyte function [194,195].

The expandability framework must be integrated with precision nutrition approaches. Individuals with low expandability may require tailored dietary interventions (lower total energy, higher protein, specific fatty acid profiles) than those with high expandability, who may tolerate more dietary flexibility [196]. Sex-specific considerations are also critical: estrogen upregulates PPARγ expression and promotes healthy SAT expansion, while postmenopausal estrogen loss is associated with VAT accumulation, increased fibrosis, and metabolic deterioration [193]. Sexual dimorphism greatly influences adipose tissue remodeling, with females demonstrating more hyperplasia (increase in adipocyte number) whereas males are characterized by more adipocyte hypertrophy [197]. Age-specific responses also matter: ASC senescence may progressively increase in older age groups, potentially reducing hyperplastic recruitment capacity. Older individuals with obesity are therefore at higher risk of MUO and may benefit from earlier, targeted weight management [193]. Beyond direct nutritional strategies, integrating structured exercise is crucial; physical activity promotes microvascular angiogenesis and mitigates extracellular matrix stiffness, thereby synergistically supporting the aforementioned dietary interventions [198]. Resistance training may be particularly beneficial because it increases muscle mass, which provides an additional oxidative sink for FFAs, reducing overflow pressure on AT [199].

Beyond conventional interventions, the expandability framework opens avenues for innovative therapeutic strategies, which must be categorized by their translational readiness. Recent advances in nanomedicine have yielded promising results for targeting AT fibrosis directly, though these approaches remain strictly experimental and preclinical. For instance, in an animal study, researchers have engineered lipid-coated mesoporous silica nanoparticles (LCMSNs) conjugated with anti-ITGA7 antibodies to selectively target a pro-inflammatory, pro-fibrotic subpopulation of ASCs expressing high levels of integrin alpha 7 (ITGA7high), demonstrating reduced AT inflammation in obese mice [200]. Similarly, retinoid-derivative lipid nanoparticles developed as part of the “fibrosis overexpression and retention” strategy have been designed to enhance mRNA expression in fibrotic regions and promote the retention of therapeutic proteins within the extracellular matrix. This approach demonstrated superior therapeutic efficacy in clinically relevant animal models of MASH and holds promise for fibrotic diseases beyond the liver [201]. In the pharmacological pipeline, dual GLP-1/glucagon receptor agonists (e.g., G49) have demonstrated favorable results in preclinical rodent models, triggering inter-organ metabolic rewiring [202]. Other mechanisms, such as PPARγ activation (e.g., pioglitazone), are backed by established interventional human clinical evidence, while FGF21 analogs (e.g., PF-05231023) exhibit anti-fibrotic and pro-adipogenic effects in preclinical models; FGF21 analogs, for instance, promote the “browning” of WAT by inhibiting mitophagy, thereby increasing energy expenditure [203,204]. In addition, while LOX inhibitor β-aminopropionitrile (BAPN) has been shown to reduce AT fibrosis, correct adipocyte-size distribution, and attenuate body weight gain in rodent models of diet-induced obesity, its clinical toxicity makes it difficult for therapeutic translation in humans [205]. Clinical trials combining bariatric surgery with such agents are warranted to determine whether these novel nanocarrier and pharmacological strategies can safely and effectively enhance expandability and improve long-term metabolic outcomes in patients with obesity.

In summary, the expandability framework provides a roadmap for integrating nutrition, exercise, pharmacotherapy, and surgery into a personalized approach to obesity-related metabolic disease. Realizing this vision will require interdisciplinary collaboration, novel biomarker development, large-scale longitudinal studies, and the translation of emerging nanocarrier and pharmacological strategies into clinical practice.

## 8. Conclusions

Adipose tissue expandability is a central biological mediator linking nutrition to metabolic disease risk. The capacity to safely store excess energy in SAT—determined by the balance between hyperplastic recruitment of new adipocytes and hypertrophic enlargement of existing cells—varies substantially among individuals. This variation is shaped by dietary composition, early-life nutrition, physical activity, genetic background, and age-related changes in adipose stem cell function. When expandability is preserved, individuals may accommodate positive energy balance with fewer metabolic complications, a pattern consistent with the metabolically healthy obesity phenotype. When expandability is exceeded, due to limited hyperplastic reserve, inadequate angiogenesis, or established fibrosis, lipids are redirected to visceral depots and non-adipose tissues, where they exert lipotoxic effects. The resulting ectopic lipid accumulation drives insulin resistance, T2D, MASLD, and CVD.

Nutrition represents a primary, highly modifiable determinant of safe energy storage capacity. Dietary fat quality (unsaturated vs. saturated), carbohydrate quality (glycemic index, fiber content), and bioactive compounds (polyphenols, omega-3 fatty acids) all influence adipose tissue expandability [206,207]. Early nutritional intervention—before advanced fibrosis and adipose stem cell exhaustion restrict tissue plasticity—is critical for maintaining expandability throughout life. This narrative review highlights adipose tissue expandability as a critical biological gatekeeper linking nutritional patterns to systemic metabolic risk. Bariatric surgery serves as a profound clinical model demonstrating that while acute nutrient off-loading and subsequent tissue-level remodeling work synergistically to restore metabolic health, underlying structural barriers like fibrosis may still constrain absolute physical storage capacity. Moving forward, clinical and translational research could benefit from evaluating precision-medicine models that extend beyond traditional weight-centric metrics. Prioritizing human longitudinal studies and validating non-invasive biomarkers of adipose health—such as circulating fibrosis peptide PRO-C3 and depot-specific imaging—will be essential to personalize nutritional and pharmacological strategies before irreversible tissue failure occurs.

## Figures and Tables

**Table 1:** Nutritional strategies potentially associated with healthier adipose-tissue function.

Nutritional Strategy	Key Metabolic Benefits	Type of Evidence	Evidence Limitations
Replacing SFAs with MUFAs/PUFAs [170]	Reduces adiposity by favoring lipid oxidation over storage	Human clinical and observational evidence	Some short-term overfeeding studies show neutral results compared to long-term observational data.
Reducing glycemic load (refined grains → whole grains) [171]	Lowers VAT, reducing risk of lipid spillover	Observational human evidence	Consistent observational association, although the cross-sectional design limits causal inference.
Adequate protein intake [172,173]	Maintains muscle quality and promotes a leaner body composition	Observational human evidence	Excessive intake may theoretically promote hypertrophy over hyperplasia via IGF-1.
High fiber intake [174]	Prevents progressive VAT accumulation. Increasing fiber intake is associated with 4% reduction in VAT.	Longitudinal human cohort evidence	Longitudinal association, although the evidence is limited to youth with overweight.
Polyphenol-rich foods [175]	Attenuates insulin resistance and reduces chronic adipose inflammation	In vitro and animal evidence	Limited large-scale human clinical trials specifically targeting AT expandability.
Limiting fructose intake [176]	Prevents adipose tissue dysfunction and chronic metabolic risk	Observational human and mechanistic evidence	Impact on SAT expansion is often independent of total caloric intake.
Omega-3 supplementation [177]	Upregulates anti-inflammatory pathways in SAT and improves insulin-stimulated glucose disposal	Human clinical trial	Evaluated via gold-standard clamps and subcutaneous fat biopsies; clinical translation is restricted by the small sample size.
IF [178]	Promotes “beiging” and healthy remodeling of adipose tissue	Mechanistic evidence; human and preclinical models	While “beiging” is well-documented in rodents, human evidence for AT remodeling is still emerging.
Low-calorie & balanced diets + exercise [179,180,181,182]	Attenuates fibrotic progression and supports insulin sensitivity	Human clinical evidence	Most established strategy for metabolic health, though it may not fully reverse established fibrosis.

Abbreviations: AT: adipose tissue; VAT: visceral adipose tissue; SAT: subcutaneous adipose tissue; SFA: saturated fatty acids; MUFAs: monounsaturated fatty acids; PUFAs: polyunsaturated fatty acids; SCFAs: short-chain fatty acids; IF: intermittent fasting; IGF-1: insulin-like growth factor 1.

## Data Availability

No new datasets were generated or analyzed in this study. All data discussed in this review were obtained from previously published studies cited in the article.

## Abbreviations

ASCs	adipose stem cells
AT	adipose tissue
ATMs	adipose tissue macrophages
BMI	body mass index
BS	bariatric surgery
ECM	extracellular matrix
ER	endoplasmic reticulum
FFAs	free fatty acids
HDL	high-density lipoprotein
IL-6	interleukin-6
IR	insulin resistance
IRS	insulin receptor substrate
LOX	lysyl oxidase
MUO	metabolically unhealthy obesity
MASLD	Metabolic dysfunction-associated steatotic liver disease
NAFPD	non-alcoholic fatty pancreas disease
NETs	neutrophil extracellular traps
NHANES	National Health and Nutrition Examination Survey
NWO	normal-weight obesity
PRO-C3	N-terminal propeptide of collagen III
PUFAs	polyunsaturated fatty acids
SAT	subcutaneous adipose tissue
SFAs	saturated fatty acids
T2D	type 2 diabetes
TNF-α	tumor necrosis factor-alpha
Tregs	regulatory T cells
VAT	visceral adipose tissue
WAT	white adipose tissue
